# Effect of immunomodulation with anti-CD40L antibody on adenoviral-mediated transgene expression in mouse anterior segment

**Published:** 2008-01-09

**Authors:** J. Cameron Millar, Iok-Hou Pang, Wan-Heng Wang, Yu Wang, Abbot F. Clark

**Affiliations:** Alcon Research, Ltd., Fort Worth, TX

## Abstract

**Purpose:**

Gene transduction using adenoviral vectors is an important research tool. To assess and optimize this technique for glaucoma research, we characterized green fluorescent protein (GFP) expression in the mouse eye after intraocular injection of adenoviral vector encoding GFP (Ad5.CMV-GFP) and evaluated the effect of anti-CD40L antibody administration on GFP expression.

**Methods:**

Mice were injected with Ad5.CMV-GFP intracamerally (IC) or intravitreally (IVT) with or without anti-CD40L antibody treatment. GFP expression was assessed by in vivo fluorescence intensity with a standardized grading scale. Location of expression was analyzed histologically by fluorescence microscopy.

**Results:**

Intraocular injection of Ad5.CMV-GFP induced titer-dependent expression of GFP in the anterior segment. In vivo fluorescence was detectable but low after IC injection. After IVT injection, fluorescence in the eye peaked at days 4–7 with a fluorescence grade of 3.0 ±0.0 (mean ±SEM, n=6; injection with 1x10^8^ pfu vector). After day 7, GFP expression declined significantly. Treatment with anti-CD40L antibody increased fluorescence intensity after IC injection, and prolonged GFP expression in the IVT group. At day 43, fluorescence grades of the IVT group were 2.8 ±0.7 (with anti-CD40L) and 1.2 ±0.6 (without antibody). Three-Way ANOVA confirmed that GFP expression was significantly higher in the anti-CD40L than the no antibody group (p=0.013), significantly higher in the IVT than the IC group (p=0.003), and significantly higher in the high viral titer (1x10^8^ pfu) than the low titer (1x10^7^ pfu) group (p=0.010). Fluorescence microscopic examination of cross-sections of eyes indicated GFP expression in the trabecular meshwork (TM), corneal endothelium, and sporadically iris, ciliary body and lens epithelium.

**Conclusions:**

Intraocular injection of Ad5.CMV-GFP induced GFP expression in the mouse anterior segment, including the TM. Expression was more prominent after IVT injection than IC injection. Anti-CD40L antibody treatment increased both intensity and duration of GFP expression. These findings provide important and practical means to improve duration and efficiency of adenovirus-mediated transgene expression in the eye.

## Introduction

Glaucoma is a sight-threatening and potentially blinding condition that affects about 70 million globally [1,2]. The molecular basis of the pathogenesis of this disease remains unclear. Its major risk factor, however, is ocular hypertension due to an abnormal reduction of aqueous humor outflow. The literature is replete with studies concerning the regulation of aqueous outflow using a variety of agents [3]. In consequence there has been much attention in the design of relevant models of glaucoma, which may then be used to understand the pathogenesis of the disease and to test potential new drugs for their ability to lower intraocular pressure (IOP). Of special interest is the development of animal models in which specific genes known to be associated with glaucoma, for example, myocilin mutants [4] and transforming growth factor β2 [5], are introduced in ocular tissues.

To this end, viral vectors have been used increasingly for the delivery of specific transgenes encoding a variety of gene products. There are many reports describing the ocular use of different vectors, such as adenovirus, adeno-associated virus, herpes simplex virus, and lentivirus [6]. In particular, viral transduction of the trabecular meshwork (TM), a structure intimately involved in the regulation of aqueous outflow, has been reported with adenovirus, which exhibits both good tropism for this tissue and relatively strong transgene expression [7-10]. Moreover, ocular transduction using adenoviral vectors does not produce observable untoward effects, nor does it nonspecifically affect aqueous hydrodynamics [9]. Unfortunately, however, the adenovirus-mediated transgene expression is transient and only lasts about 1 to 2 weeks [7,8], thus limiting its usefulness as a research tool in long-term studies.

The transient nature of adenoviral expression is likely due to an immune response generated against viral proteins and/or transgene products [8,11,12]. Hence, various strategies have been designed to prolong viral expression via immunosuppression. One of these strategies involves blockade of the binding of CD40 to CD40 ligand (CD40L, also known as CD154). CD40 is a costimulatory protein found on antigen presenting cells. Binding of CD40 to CD40L on T-lymphocytes leads to activation of the antigen presenting cells and T-cells, and produces a variety of downstream effects, including both humoral and cellular immune responses [13-15]. This interaction can be effectively impeded by treatment with the anti-CD40L antibody, which has been reported to be effective as an adjunctive immunomodulating agent for facilitation of organ transplantation, as well as treatments for atherosclerosis, systemic lupus erythromatosus and rheumatoid arthritis [16]. Most importantly, systemic treatment of mouse with anti-CD40L antibody significantly extends the duration of transgene expression of recombinant adenovirus in the liver [17-19].

In this study, we characterized the expression profile and duration of an adenoviral vector (Ad5.CMV-GFP) encoding jellyfish (*Aequorea victoria*) green fluorescent protein (GFP) after intracameral (IC) or intravitreal (IVT) injection of different titers. More importantly, we also determined whether transgene expression in the mouse eye could be prolonged via treatment with anti-CD40L antibody.

## Methods

### Animal husbandry

All animal experiments were conducted in compliance with the ARVO Statement for the Use of Animals in Ophthalmic and Vision Research, and the Alcon Animal Care and Use Committee regulations. Adult male Balb/cJ mice (Jackson Laboratory, Bar Harbor, ME) weighing between 20 and 30 g (three to five months of age) were used in this study. The animals were maintained on a 12 h light/12 h dark cycle in a temperature-controlled (22 °C) facility. Food and water were available *ad libitum.*

### Ad5.CMV-GFP

Ad5.CMV-GFP was purchased from Qbiogene (Montreal, Canada). This viral vector is a first generation (ΔE1/ΔE3) adenovirus serotype 5 particles expressing the GFP gene under control of the cytomegalovirus-IE promoter/enhancer. The expression of GFP can be easily monitored by the green fluorescence. Therefore, it is used extensively as a marker to test the infectivity of specific cells both in vitro and in vivo.

### Intraocular injection

Mice were examined at day −1 by direct ophthalmoscopy (Hand-Held Ophthalmoscope, Welch-Allyn, Model 11710, Skaneateles Falls, NY) to confirm a normal appearance, free of any signs of ocular disease. At day 0, mice were anesthetized with an intraperitoneal injection of an anesthesia cocktail (ketamine, 73 mg/kg, Fort Dodge, IA; xylazine, 1.8 mg/kg, Vetus, Burns Veterinary Supply, Westbury, NY; acepromazine, 1.8 mg/kg, Vetus). The right eye (OD) of each animal was then topically anesthetized with one to two drops of 0.5% proparacaine (Alcaine®, Alcon Laboratories, Fort Worth, TX) and then given a single intravitreal (IVT) or intracameral (IC) injection of a suspension of Ad5.CMV-GFP (1x10^7^ pfu or 1x10^8^ pfu in a volume of 2 μl). Eyes given IC injection were also pretreated with one to two drops 1% cyclopentolate (Mydriacyl®, Alcon Laboratories) to dilate the pupil. Ocular injections were administered using a Hamilton glass microsyringe (10 μl capacity) fitted with a custom-made one-inch 33G needle with a 10° bevel (Hamilton, Reno, NV).

### Immunomodulation

Hamster anti-mouse CD40L antibody (Biosource International, Camarillo, CA) was used to suppress immune responses by blockade of the interaction between CD40 and CD40L. Assigned animals were injected with this antibody (0.5 mg/mouse, intraperitoneal) on days −1, 0 (immediately preceding intraocular injection), 1, 2, 5, 9, and 14, a treatment schedule modified from that reported by Stein et al. [18]

### In vivo evaluation

Commencing on day 4 after the injection and then on following predetermined days, each injected eye was examined under ultraviolet illumination (Illumatool® Model LT-9900; Lightools Research, Encinatas, CA) through a filter (excitation 470 nm, emission 515 nm) for fluorescence, as an indication of presence of GFP in the anterior chamber. Based on its intensity and distribution, fluorescence grades were assigned at the indicated days by a researcher who was masked to the treatments. Throughout the study, all eyes (injected and control) were carefully examined with a hand-held ophthalmoscope for the presence of iridial hyperemia and lenticular opacity. Animals were also assessed daily for potential gross abnormalities in appearance and behavior by the veterinary staff.

### Histological analysis

Following assessment of fluorescence and ophthalmic examination on days 6 and 43, randomly assigned animals were euthanized (CO_2_ asphyxiation). Both eyes, injected and uninjected control, were enucleated and fixed with 4% paraformaldehyde. Eyes were embedded with Optimal Cutting Temperature (OCT) embedding medium and stored at −80 °C until cryosectioned (thickness=10 μm). The sections were evaluated by fluorescence microscopy for evidence of GFP expression in various ocular tissues.

### Statistical analysis

Fluorescence grades were expressed as mean ±SEM. Three-way ANOVA was used to analyze the effects of injection routes, viral titers, and antibody treatment on the integrated fluorescence grades. P values of less than 0.05 were regarded as statistically significant.

## Results

### In vivo fluorescence grading scale

**Figure 1:**
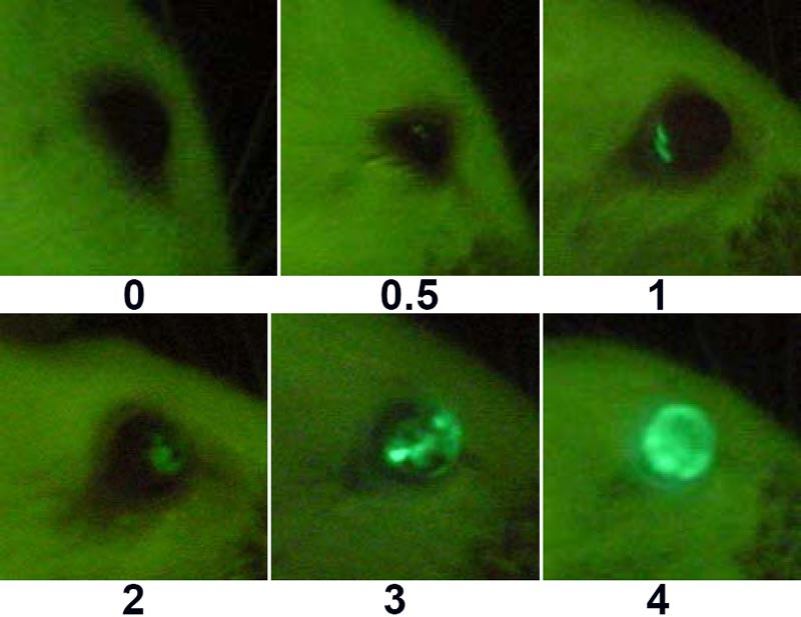
Representative examples of mouse eyes with various fluorescence grades. The number under each image indicates the assigned grade. Definitions of the grades are listed in Table 1.

To assess the in vivo expression of GFP, we designed a standardized, semi-quantitative ordinal fluorescence grading scale based on the intensity and diffusiveness of the observed fluorescence in the mouse eye. This scale ranges from 0, indicating no observable fluorescence, to 4, which represents brilliant fluorescence throughout the anterior segment (Table 1). According to this grading system, eyes of grade 0.5 or 1 had small discrete spots of barely or readily, respectively, detectable fluorescence typically only in the region of the chamber angle. Eyes of grades 2 and 3 expressed GFP in a much larger portion of the chamber angle as well as other structures of the anterior segment. Figure 1 demonstrates images of representative eyes with the respective grades.

**Table 1:** Definitions of in vivo fluorescence ordinal grading scale.

**Grade**	**Observations**
0	No visible fluorescence
0.5	Barely visible small discrete spot(s) of fluorescence, typically starts in the region of the chamber angle
1	Readily visible small discrete spot(s) of fluorescence, typically in the region of the chamber angle
2	Larger patches of fluorescence in the chamber angle and some patches in the cornea but significant areas of the anterior segment are still not fluorescing
3	Entire visible area of anterior chamber, lens, cornea, and iris dimly to somewhat brightly fluorescing
4	Entire visible area of anterior chamber, lens, cornea, and iris brilliantly fluorescing

This standardized semi-quantitative grading scale was used to assess the in vivo expression of GFP in the anterior segment of mouse eyes.

### Expression of GFP: in vivo observations

**Figure 2:**
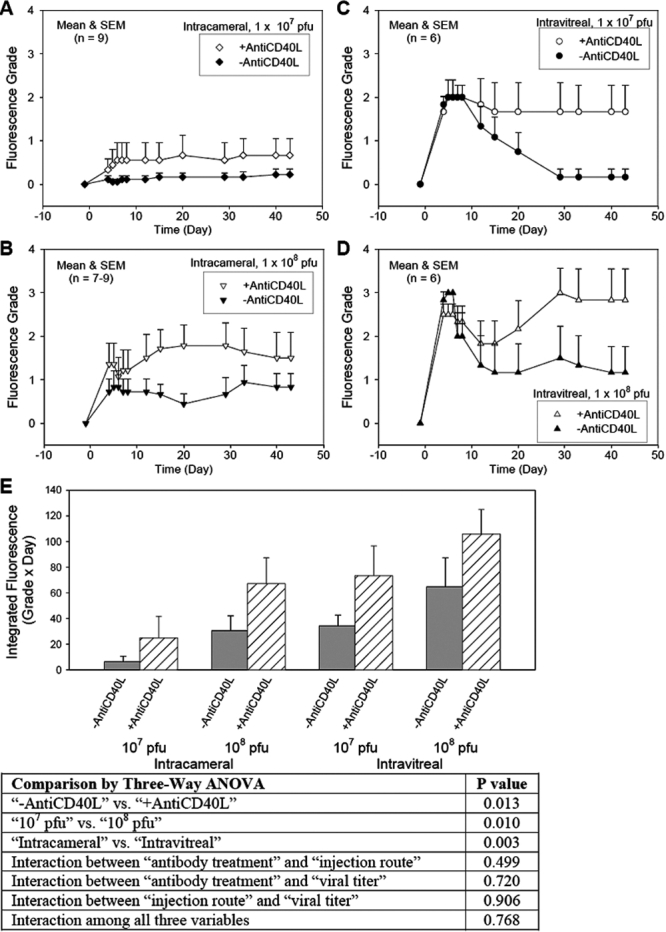
Effects of routes of intraocular injections, titers of viral vector, and use of anti-CD40L antibody on the intensity and duration of GFP expression evaluated by in vivo fluorescence grades. At day 0, Ad5.CMV-GFP, at a titer of 1x10^7^ pfu (**A** and **C**) or 1x0^8^ pfu (**B** and **D**), was injected intracamerally (**A** and **B**) or intravitreally (**C** and **D**) with (open symbols) or without (closed symbols) immunomodulation by intraperitoneal treatment of anti-CD40L antibody. In vivo fluorescence in the eyes was evaluated and fluorescence grades assigned at the indicated days by a researcher who was masked of the treatments. The fluorescence grades were further analyzed as the integrated fluorescence (**E**) by summation of the area under the curve of each animal. All data were presented as mean and SEM.

**Figure 3:**
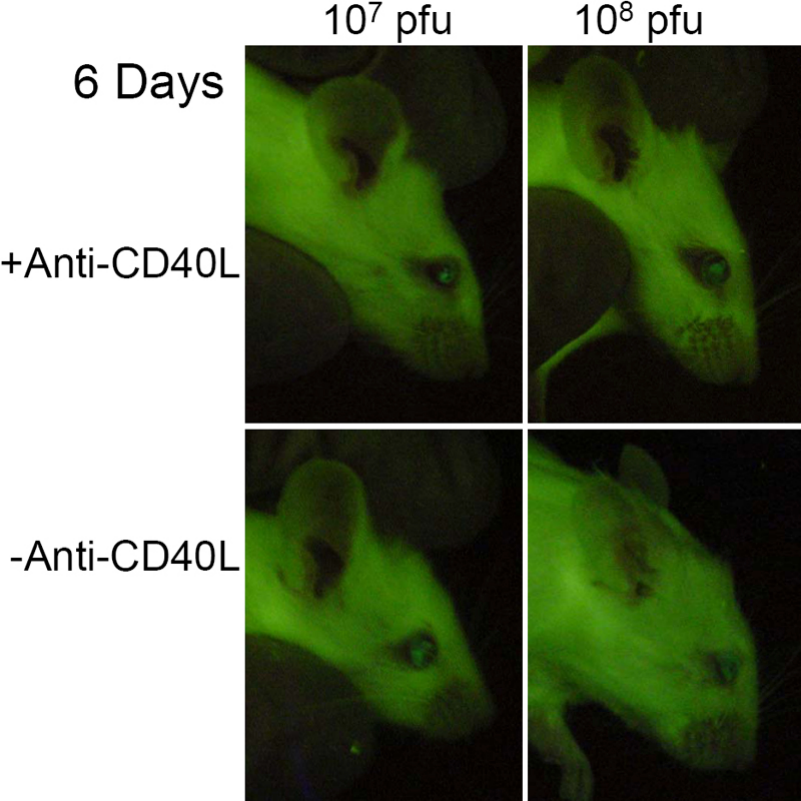
GFP fluorescence in mouse eyes six days following intravitreal injection. Images of mouse eyes six days after intravitreal injection of Ad5.CMV-GFP at 1x10^7^ pfu (left panels) or 10^8^ pfu (right panels) with (upper panels) or without (lower panels) anti-CD40L treatment. All eyes were expressing a substantial level of GFP with fluorescence grades of 2 or 3.

Intraocular injections, both IC and IVT, of Ad5.CMV-GFP induced production of GFP and hence fluorescence in the anterior segment of the mouse eye. The intensity and duration of GFP expression were dependent on the injection route, viral titer, and treatment with antiCD40L antibody. For example, IC injection of 1x10^7^ pfu without antibody treatment produced a barely detectable fluorescence in the anterior segment (fluorescence grade at day 5=0.06 ±0.06, mean ±SEM, n=9; Figure 2A). Whereas, increasing the injected viral titer to 1x10^8^ pfu boosted GFP expression to a higher level (Fluorescence Grade at day 5=0.83 ±0.38, n=9; Figure 2B). Interestingly, when Ad5.CMV-GFP was injected intravitreally, the expression of GFP in the anterior segment was clearly higher than when it was injected intracamerally. Specifically, IVT injection of Ad5.CMV-GFP at 1x10^7^ pfu produced a peak fluorescence intensity at days 4–7, with a fluorescence grade at day 5 of 2.00 ±0.00 (n=6; Figure 2C and Figure 3). Similar to IC injection, IVT injection of the viral vector at a higher titer (1x10^8^ pfu) generated higher fluorescence expression (fluorescence grade at day 5=3.00 ±0.00 [n=6; Figure 2D and Figure 3]). The effect in the IVT groups however began to diminish starting at day 7. By day 30 fluorescence had almost disappeared in both the IC and IVT groups injected with the lower titer (1x10^7^ pfu) of Ad5.CMV-GFP (Figure 2).

**Figure 4:**
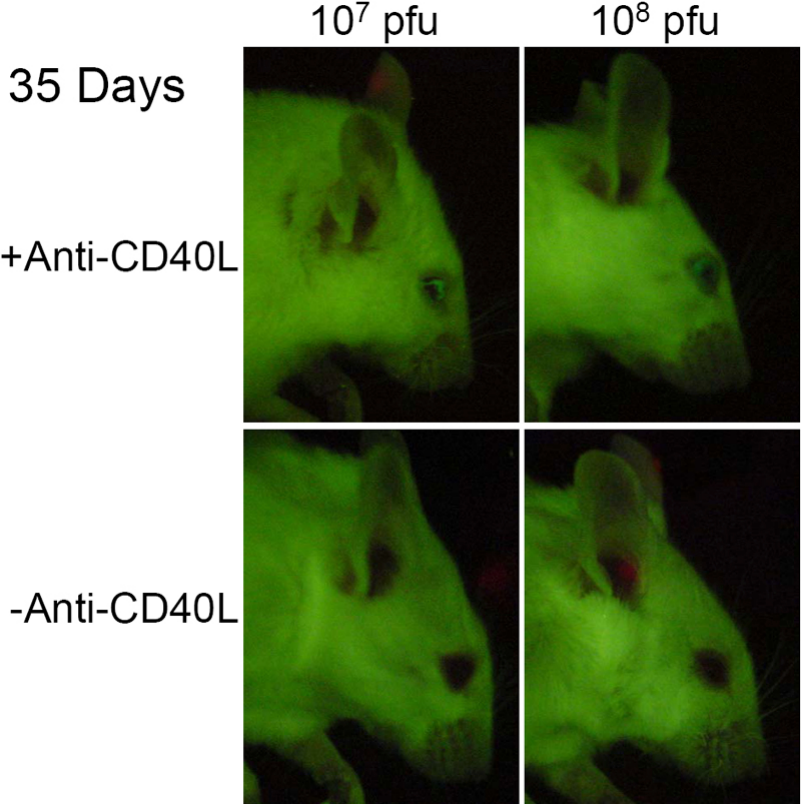
GFP fluorescence in mouse eyes 35 days following intravitreal injection. Images of mouse eyes 35 days after intravitreal injection of Ad5.CMV-GFP at 1x10^7^ pfu (left panels) or 10^8^ pfu (right panels) with (upper panels) or without (lower panels) anti-CD40L treatment. The eyes from mice treated with anti-CD40L antibody still expressed high level of GFP; their fluorescence grades were between 2 and 3. In contrast, eyes from mice without anti-CD40L treatment had minimal fluorescence with grades of 1 or below.

Regardless of injection routes or titers of Ad5.CMV-GFP used, immunomodulation with treatment of anti-CD40L antibody always enhanced GFP expression in the mouse eye. After IC injections, anti-CD40L antibody treated animals showed an increase in fluorescence intensities. For example, the fluorescence grade at day 5 of Ad5.CMV-GFP (1x 10^7^ pfu)-transfected eyes increased to 0.44 ±0.36, while that of 1x10^8^ pfu-injected eyes increased to 1.36 ±0.48. The elevated fluorescence persisted or was further raised slightly until at least day 43 (Figure 2). The anti-CD40L antibody did not significantly affect the peak fluorescence level in the IVT group, such that the fluorescence grades at day 5 of eyes injected intravitreally with either 1x 10^7^ pfu or 1x10^8^ pfu did not differ with or without antibody treatment. However, antibody treatment dramatically lengthened the duration of GFP expression in these animals. Without anti-CD40L antibody, IVT injection of 1x10^7^ pfu Ad5.CMV-GFP produced a fluorescence grade of 0.17 ±0.18 at day 43. Antibody treatment augmented that to 1.66 ±0.61 (Figure 2C). Likewise, anti-CD40L antibody improved fluorescence grade at day 43 from 1.17 ±0.59 to 2.83 ±0.72 in the 1x10^8^ pfu group (Figure 2D). Representative images at day 35 of intravitreally injected mice are shown in Figure 4.

The effects of injection routes, viral titers, and antibody treatment on ocular GFP fluorescence can be summarized by the integrated fluorescence grades. These values were obtained by integrating the area under the “Fluorescence Grade” versus “Time” curve of each animal. As seen in Figure 2E, it is obvious that the integrated fluorescence grade was affected by the injection route, viral titer injected, and the use of anti-CD40L antibody. This observation was confirmed by analysis of the data with three-way ANOVA, which indicated that GFP expression was significantly higher in the anti-CD40L than the no antibody group (p=0.013), significantly higher in the IVT than the IC group (p=0.003), and significantly higher in the high viral titer (1x10^8^ pfu) than the low titer (1x10^7^ pfu) group (p=0.010). Furthermore, there was no statistically significant (p>0.05) interaction between any two or among all three variables.

Intraocular injection of viral vectors did not cause any abnormalities in the majority (57%) of eyes as observed by ophthalmoscopic examination throughout the study period. Among the vector-injected eyes, at day 6 after injection, 26% were found to develop iridal hyperemia: 23% had slight but detectable redness or dilation of iridal vessels, whereas 3% displayed moderate redness of the whole iris. Lenticular opacity was noted in 21% of the injected eyes: 9% had barely visible or very small discrete opacity, and 12% had opacity that involved more than 20% of the visible area of the lens. These untoward effects typically appeared between days 4 and 6 after injection, and persisted till end of study. There was no correlation between the untoward effects and injection routes, viral titers, antibody treatment, or fluorescence grades. None of the treatments produced any systemic untoward effects as judged by daily evaluation of gross appearance and behavior of the animals.

### Expression of GFP: fluorescence microscopy

**Figure 5:**
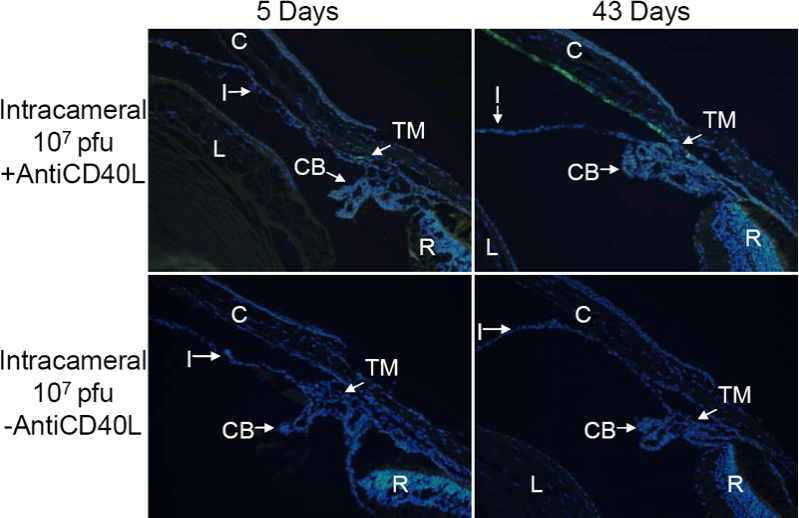
Representative fluorescence photomicrographs of cross-sections of mouse anterior segments treated with intracameral injection of Ad.CMV-GFP (1x10^7^ pfu). The animals were euthanized at either 5 or 43 days after vector injection with or without anti-CD40L antibody treatment. Green fluorescence=GFP, blue fluorescence=DAPI staining of cell nuclei. The occasional yellowish-green fluorescence represents auto-fluorescence of certain tissues, such as those in the outer nuclear layer and retinal pigmented epithelium, which was also observable in non-injected eyes (Data not shown). Abbreviations: C=cornea; CB=ciliary body; I=iris; L=lens; R=retina; TM=trabecular meshwork.

**Figure 6:**
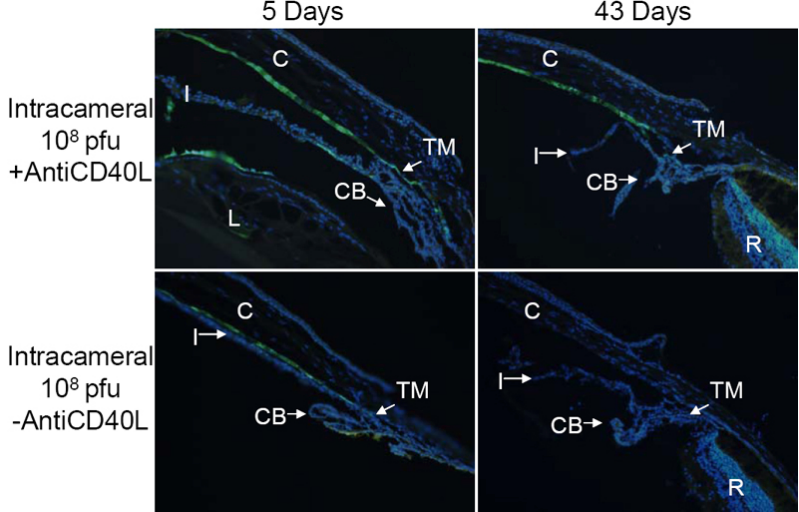
Representative fluorescence photomicrographs of cross-sections of mouse anterior segments treated with intracameral injection of Ad.CMV-GFP (1x10^8^ pfu). Representative fluorescence photomicrographs of cross-sections of mouse anterior segments treated with intracameral injection of Ad5.CMV-GFP (1x10^8^ pfu). See legend of Figure 5 for additional information.

Cross-sections of mouse eyes obtained from animals euthanized at days 5 and 43 following intraocular Ad5.CMV-GFP injection were examined by fluorescence microscopy, and GFP expression was typically localized to the cornea, TM, iris, ciliary body, and lens. No GFP fluorescence was detected in the retina regardless of injection route. Furthermore, the observed ocular distribution and intensity of GFP expression correlated very well with in vivo fluorescence grades of the different groups. For example, not much fluorescence in the anterior segment was observed at 5 and 43 days after IC injection of 1x10^7^ pfu Ad5.CMV-GFP without anti-CD40L antibody treatment (Figure 5), while treatment with the anti-CD40L antibody enhanced the expression of GFP of the same injection regimen. Under this condition, a fraction of cells in the TM and corneal endothelium expressed GFP (Figure 5). IC injection at a higher titer of the vector (1x10^8^ pfu), even without anti-CD40L antibody treatment, produced more noticeable GFP expression. At day 5, fluorescence was apparent in the corneal endothelium, TM and the ciliary body. However, the GFP fluorescence diminished and was barely visible in eyes 43 days after injection (Figure 6). Again, treatment with anti-CD40L antibody augmented the expression of GFP. In eyes 5 and 43 days after IC injection of 1x 10^8^ pfu Ad5.CMV-GFP, the TM and corneal endothelium, and sporadically the lens epithelium and iris were positive with GFP fluorescence (Figure 6).

**Figure 7:**
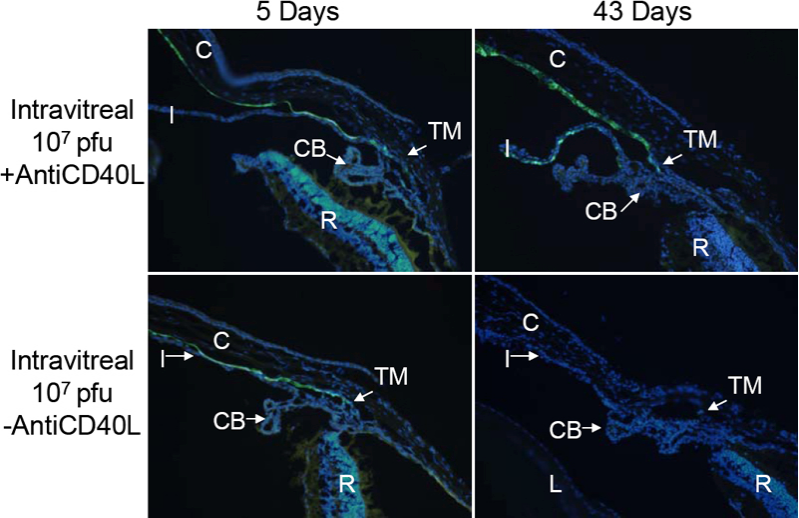
Representative fluorescence photomicrographs of cross-sections of mouse anterior segments treated with intravitreal injection of Ad.CMV-GFP (1x10^7^ pfu). Representative fluorescence photomicrographs of cross-sections of mouse anterior segments treated with intravitreal injection of Ad5.CMV-GFP (1x10^7^ pfu). See legend of Figure 5 for additional information.

**Figure 8:**
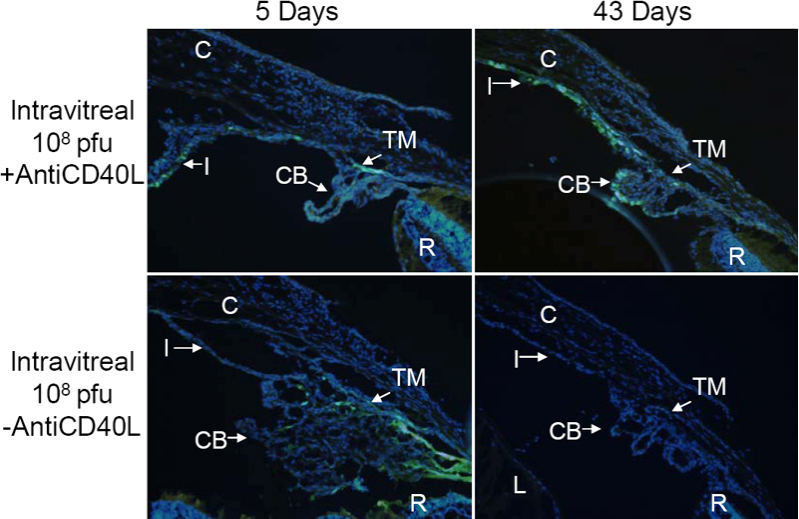
Representative fluorescence photomicrographs of cross-sections of mouse anterior segments treated with intravitreal injection of Ad.CMV-GFP (1x10^8^ pfu). Representative fluorescence photomicrographs of cross-sections of mouse anterior segments treated with intravitreal injection of Ad5.CMV-GFP (1x10^8^ pfu). See legend of Figure 5 for additional information.

As indicated by the in vivo fluorescence grades, IVT injection produced more efficient expression of the injected GFP vector than IC injection. Fluorescence microscopic observation substantiated this conclusion. At day 5 after IVT injection of 1x10^7^ pfu Ad5.CMV-GFP, with or without anti-CD40L antibody treatment, significant fluorescence was noted in the TM, corneal endothelium, and occasionally the iris (Figure 7). With the anti-CD40L treatment, a similar distribution of fluorescence was still present at day 43, whereas in animals without anti-CD40L antibody, minimal fluorescence was detected at 43 days after injection (Figure 7). Higher titer of the vector produced more prominent GFP expression. Thus, five days after IVT injection of 1x10^8^ pfu, the TM, corneal endothelium, and sometimes the iris, lens epithelium and ciliary body demonstrated fluorescence (Figure 8). Treatment with anti-CD40L antibody prolonged this expression profile till at least 43 days after vector injection, while without the antibody, fluorescence faded and became faint at day 43 (Figure 8).

Therefore, the histological examination of ocular tissues after various treatment procedures confirmed the observations by in vivo fluorescence, in that anti-CD40L antibody treatment increased the intensity of ocular GFP expression after IC injection and prolonged its duration of expression after IVT injection. Moreover, the fluorescence microscopic assessments also supported the notions that expression of GFP depended on the titers used and that IVT injection produced more efficient expression than IC injection.

## Discussion

We describe in this study that Ad5.CMV-GFP, when delivered by intraocular (IC or IVT) injection into the eye of the Balb/cJ mouse, led to GFP expression in the anterior segment structures. In vivo visual examination of the eyes under UV illumination and fluorescent microscopic observation of ocular cross-sections indicated GFP expression in the region of the TM, as well as other tissues, particularly the corneal endothelial cells, and occasionally the iris epithelium, ciliary body, and lens epithelium. The intensity and extensiveness of distribution of GFP expression depended on the viral titer used. The higher viral titer (1x10^8^ pfu) yielded consistently and significantly higher GFP expression than the lower titer (1x10^7^ pfu).

We found that eyes given IVT injections yielded reliably better GFP expression than those given IC injections, probably because relatively rapid washout of injected viral vector following IC injection resulted in a low effective viral concentration in the anterior chamber, whereas IVT injection led to a longer-lasting depot in the vitreous. This finding agrees with our experience that IVT injection of adenoviral vectors encoding various transgenes produces more pronounced expression in the TM and other cells in the anterior segment than those induced by IC injections (unpublished observations). Thus, IVT injection of adenovirus containing specific myocilin mutants generated significant ocular hypertension in the mouse [4]. Interestingly, even though the vitreous is in close proximity to the inner retina, we could not detect GFP fluorescence in retinal tissues after IVT injection of Ad5.CMV-GFP. Others have reported that IVT injection of adenoviral vectors produced transgene expression in the retina. For example, DiPolo et al. [20] demonstrated adenoviral vector-induced expression of brain-derived neurotrophic factor in Müller cells. The discrepancy between these results and the current data are likely due to the lower sensitivity of fluorescence as a detection means compared to methods such as immunohistochemistry. Regardless, our findings suggest that adenoviral vectors have a lower tropism for cells in the inner retina compared to cells in the TM. Our results are broadly in keeping with those of Budenz et al. [7], who also compared the expression profile of IVT and IC injections of adenovirus encoding LacZ in mice.

Similar to previously published reports [7,8], our adenovirus-mediated transgene expression in the mouse eye was transient, most probably a result of an immune response generated against viral proteins [8,11,12]. We reduced this shortcoming by immunomodulation; treating the animals with the anti-CD40L antibody, which clearly increased the expression of GFP after IC injection, and significantly prolonged GFP expression after IVT injection to more than six weeks. In fact, our most recent data suggest that ocular transgene expression could be lengthened to at least 12 weeks by this antibody (unpublished observations). These observations concur with prior findings that treatment of mouse with this antibody significantly extended the duration of adenoviral transgene expression in the liver [17-19].

Even though the anti-CD40L antibody is an immunomodulating agent, the treated animals did not exhibit any signs of infection or compromised health. In all respects they appeared to be completely normal and healthy over the entire course of the experiment, showing no noticeable alterations in behavior or food or water consumption. The lack of general untoward effects of the anti-CD40L antibody is consistent with its relatively specific suppression of the CD4^+^ T-cell-assisted but B-cell-mediated humoral responses normally mounted against adenovirus, without having a large number of other adjunctive actions [13-15]. Indeed, anti-CD40L antibody has been administered in mice [17-19,21], rats [22], baboons [23,24], and monkeys [25-27] with minimal undesirable consequences.

Therefore, treatment with anti-CD40L antibody represents a practical and convenient method to improve and prolong adenoviral transgene expression in the anterior segment of the mouse eye. Hoffman et al. [8] were similarly successful in lengthening the duration of ocular expression by injecting the adenovirus into immunodeficient (*nu*/*nu*) CD-1 mice. Nonetheless, using the *nu*/*nu* CD-1 mouse may not be a practical solution for many ophthalmological studies. The same researchers also demonstrated that CTLA4Ig, an immunoglobulin shown to block the interaction of CD80 and CD86 ligands on antigen-presenting cells with CTLA4 receptors on T-cells, appeared to stabilize the expression of adenovirus in the TM [8]. The effect of this treatment, however, was not significantly different from that of treatment with a control immunoglobulin.

The extension of adenoviral transgene expression in tissues of the anterior segment, such as the TM, is an important accomplishment. Other vector systems have been used to deliver genes to this region in various animals, for example, adeno-associated virus [6], herpes simplex virus [28,29], lentivirus [30,31], and liposomes [32]. Some of these methods, such as adeno-associated virus [6] and liposomes [32], seemed ineffective. Herpes simplex viral vectors were capable of efficient gene delivery to structures in the anterior segment. However, in the reported studies, expression of the encoded gene lasted only 10 days [28,29]. The exact mechanism of this transience remains unknown, but a promoter shutoff may be involved. Lentiviral vectors were successfully applied to deliver genes to the TM [30,31]. An important advantage of these vectors is their ability to integrate into the host cell genome even in slowly dividing or non-dividing cells, and to induce long-term transgene expression. Nevertheless, a key disadvantage of this approach is the potential for insertional mutagenesis, which, albeit uncommon, can present considerable challenges to data analysis and interpretation. Hence, adenoviral vectors still serve a vital function in gene delivery to the anterior segment in various ophthalmological studies, particularly those related to glaucoma research. Indeed, among all the different vectors, it is one of the most popular and has been productively used to introduce transgenes into the TM of mice [7,8], rats [33], rabbits [34], dogs [35], and monkeys [36], Our method of immunomodulation significantly improves its expression duration and overcomes the major limitation of this gene delivery system.

In summary, we found that GFP can be introduced into the anterior segment of the Balb/c mouse via a single IVT or IC injection of Ad5.CMV-GFP. Appropriate titers for this adenoviral vector appeared to be between 1x10^7^ and 1x10^8^ pfu, and IVT injection was the better method of delivery. GFP was expressed in the TM, corneal endothelium, and to some extent other anterior segment tissues. Immunomodulation of the animals by intraperitoneal injection of anti-CD40L antibody enhanced both the time and intensity of GFP expression.
